# Cardiovascular risk goal attainment in the United States rheumatologic population

**DOI:** 10.1016/j.ajpc.2026.101611

**Published:** 2026-04-06

**Authors:** Kaitlyn Granstaff, Sarah Jiang, Sanjana Nallagatla, Anna Grodzinsky

**Affiliations:** a University of Missouri Kansas City School of Medicine, Kansas City, MO, USA; b Saint Luke’s Health System/Saint Luke’s Mid America Heart Institute, USA; c Saint Luke’s Muriel Kauffman Institute for Women’s Cardiovascular Research, Kansas City, MO, USA

**Keywords:** Primary prevention, Cardio-rheumatology, lipid management

## Abstract

**Background:**

Rheumatologic diseases are independently linked to premature cardiovascular (CV) events. The emerging field of cardio-rheumatology addresses the intersection of inflammation and accelerated atherosclerosis driving excess CV risk. Despite calls to action, data on risk factor optimization, antihyperlipidemic therapy, and comorbidities remain limited. Using the largest US analytic cohort to date, we examined antihyperlipidemic prescription patterns in patients with rheumatologic disease and elevated atherosclerotic cardiovascular disease (ASCVD) risk.

**Methods:**

We analyzed deidentified EPIC Cosmos records (277 million patients, 1,597 hospitals, 17 billion encounters) from 1/2020-1/2025. Adults with any of 11 rheumatologic conditions associated with elevated CV risk—including rheumatoid arthritis, systemic lupus erythematosus, psoriatic arthritis, ankylosing spondylitis, and systemic sclerosis—were included. We described prevalence of elevated ASCVD risk (=7.5%), prescription rates of antihyperlipidemic therapy and attainment of guideline directed LDL-C <70 mg/dL.

**Results:**

Among ≈4 million included patients, ASCVD-related comorbidities were common: diabetes (19.6%), hypertension (52.1%), dyslipidemia (51.4%). Mean LDL was 98 mg/dL; mean A1c was 7.1% in diabetics and 5.6% in nondiabetics. Only 18.1% achieved LDL <70 mg/dL during the study period. Despite 41.9% having a Cardiology and 36.4% a Rheumatology encounter, 22.7% of dyslipidemic patients were not on antihyperlipidemic therapy. Among those with ASCVD risk 7.5-20%, 28.5% were not on antihyperlipidemic therapy (17.0% with myalgias). For ASCVD risk >20%, 19.9% were not on antihyperlipidemic therapy (17.3% with myalgias).

**Conclusion:**

In this large US cohort, lipid management and CV risk goal attainment were suboptimal across rheumatologic conditions. These findings underscore the need for multidisciplinary cardio-rheumatology care and implementation strategies to improve outcomes in this high-risk population.

## 1 Background

Cardiovascular disease (CVD) is a major cause of morbidity and mortality worldwide, and patients with underlying inflammatory or autoimmune diseases have a greater risk of earlier CVD. Cardio-rheumatology is an emerging field focused on addressing CVD risk factors and sequelae in patients with rheumatological conditions [1]. This increased risk is multifactorial but partly attributable to chronic systemic inflammation, which promotes endothelial dysfunction, arterial stiffness, and accelerated atherogenesis [2]. Mitigating CVD risk in these patients is critical given cardiovascular complications are a leading cause of mortality in patients with rheumatologic diseases [3].

The 2018 American College of Cardiology (ACC) and American Heart Association (AHA) guidelines for primary prevention recommend assessing the atherosclerotic cardiovascular disease (ASCVD) risk score. Patients with high ASCVD risk (≥20%) were recommended to target blood pressures <130/80 mmHg, low-density lipoprotein cholesterol (LDL-C) <70 mg/dL, and lifestyle modifications including smoking cessation, dietary improvements, and weight management. Patients with diabetes should have a goal Hb A1C of 6.5–7% [4]. Similarly, the European Alliance of Associations for Rheumatology (EULAR) guidelines for CVD risk management recommends aggressive management of modifiable risk factors. Further, the 2026 ACC/AHA multi-society lipid guidelines encourage earlier risk-assessment and intervention, and distinct LDL-C goals of <100 mg/dL for moderate risk and <70 mg/dL for high risk [5].

Despite these recommendations, population-level data on cardiovascular risk factor control in patients with rheumatologic conditions remain limited. Using de-identified EPIC COSMOS data from January 2020 to January 2025, we evaluated patients with eleven rheumatologic diagnoses associated with higher CVD risk. Our objective was to determine the proportion of patients meeting guideline-directed CVD prevention goals as outlined by the AHA and EULAR. As such, we aimed to inform more targeted strategies to reduce risk of adverse cardiovascular outcomes by 1) examining the proportion of patients achieving primary prevention targets 2) reporting patterns of lipid-lowering therapy prescription and drug-related myopathy and 3) assessing exposure to rheumatology/cardiology specialty encounters in large, geographically representative cohort.

## 2 Methods

In this cross-sectional cohort study, we analyzed patient data from the EPIC COSMOS database (∼277 million patients, 1597 hospitals, 17 billion encounters). Because no identifiable patient data were accessed, this study received a determination of exemption per Saint Lukes’s Hospital institutional IRB. Aggregate-level analyses were conducted using the Epic Cosmos “Slicer Dicer” tool. Diagnoses were identified using Epic Cosmos groupers, which aggregate related conditions across Systematized Nomenclature of Medicine–Clinical Terms (SNOMED CT) and International Classification of Diseases, Tenth Revision (ICD-10) codes into unified clinical categories to enable consistent disease identification across institutions. When available, American College of Cardiology (ACC)–developed groupers were used to define cardiovascular comorbidities and medication classes. A comprehensive list of ICD diagnostic codes defining the above-mentioned rheumatologic conditions, as well as list of medications queried, were recorded.

Patients with a clinical diagnosis of RA, psoriasis, PsA, SLE, Sjögren’s syndrome, sarcoidosis, polymyalgia rheumatica (PMR), ankylosing spondylitis, scleroderma, antiphospholipid antibody syndrome, and giant cell arteritis (GCA) between January 23, 2020 and January 22, 2025 were included in the query.

Cardiovascular risk features assessed included ASCVD risk scores, serum lipid levels, hypertension status, glycemic control, and smoking status. The EPIC ASCVD risk estimator is based on the 2020 ACC ASCVD Risk Estimator Plus, which uses the Pooled Cohort Equation and incorporates the Million Hearts Longitudinal Model to update risk according to treatment response. Patients were stratified based on their 10-year ASCVD risk scores, with emphasis on those at high-risk (10-yr risk score ≥20%). Those with diagnoses related to major adverse cardiovascular events (MACE) were excluded in ASCVD-based primary prevention analyses. MACE was defined as diagnoses of myocardial infarction and/or cerebrovascular disease. We evaluated lipid management by assessing the proportion of patients prescribed any class of lipid-lowering therapies, including statins, PCSK9 inhibitors, cholesterol absorption inhibitors, bile acid sequestrants, adenosine triphosphate lyase inhibitors, fibrates, omega-3 supplements, and nicotinic acid. Lipid-lowering intolerance was classified per ICD-based diagnosis related to myalgias, rhabdomyolysis, myopathy, or adverse effect related to lipid-lowering agents.

Social vulnerability index, a metric calculated from US census data and reflected in the electronic medical record (EMR) at the patient-level, was also analyzed. This index is designed to reflect a geographic community’s resilience to natural or economic stressors through a series of variables including demographic and socioeconomic indices.

Statistically, we present categorical variables as counts and percentages. Tests of significance were not performed, as there was no hypothesis testing or between-group comparison.

## 3 Results

The analytic cohort included 4066,153 patients. RA was the most prevalent rheumatologic condition (1.4 million patients), with additional rheumatologic conditions, and relevant clinical and demographic characteristics listed (Table 1). The average social vulnerability index was near the projected US median of 50% across all diagnoses, reflecting relative vulnerability to geographic and socioeconomic obstacles. A score of 0 reflects the least amount of vulnerability while 100% is the most vulnerable.

**Table 1:** Demographic characteristics of analytic cohort, by rheumatologic diagnosis.

Rheumatologic Diagnosis	Female (%)	Age <40 at Time of Enrollment in COSMOS (%)	Self-Reported Race = White (%)	Current Tobacco Use (%)	Average Social Vulnerability Index* (%)
Rheumatoid Arthritis (n = 1376,104)	75.9%	9.9%	71.3%	13.2%	47.0%
Psoriasis (n = 1304,624)	55.5%	27.0%	74.2%	15.4%	43.0%
Psoriatic Arthritis (n = 373,681)	59.6%	17.3%	80.8%	13.0%	42.0%
Systemic Lupus Erythematosus (n = 369,014)	89.3%	29.4%	59.2%	12.4%	50.0%
Sjögren’s Syndrome (n = 259,128)	91.5%	14.9%	72.2%	7.1%	43.0%
Sarcoidosis (n = 257,563)	58.3%	10.7%	55.6%	9.2%	47.0%
Polymyalgia Rheumatica (n = 249,671)	60.4%	1.1%	82.9%	6.3%	39.0%
Ankylosing Spondylitis (n = 160,997)	49.6%	26.2%	77.1%	12.6%	42.0%
Antiphospholipid Syndrome (n = 137,279)	72.8%	32.0%	72.2%	12.3%	44.0%
Scleroderma (n = 94,536)	85.9%	13.0%	72.2%	8.9%	44.0%
Giant Cell Arteritis (n = 93,117)	69.4%	2.1%	75.4%	9.2%	44.0%
Total (All Diagnoses) (n = 4066,153)	67.3%	18.0%	72.5%	12.7%	45.0%

*The Social Vulnerability Index (SVI) overall estimated percentage for the patient's most recent census tract of residence, compared to the US. This value is derived from census tract data provided by the US Centers for Disease Control and Prevention (CDC). For SVI, 100 represents the most vulnerable and 0 represents the least.

Cardiometabolic comorbidities were highly prevalent (Table 2). 41.9% of patients had a Cardiology and 36.4% a Rheumatology encounter during the study period.

**Table 2:** Prevalence of cardiometabolic comorbidities by rheumatologic diagnosis.

Rheumatologic Diagnosis	Hypertension (%)	Hyperlipidemia (%)	Diabetes Mellitus - (Type 1 or Type 2) (%)
Rheumatoid Arthritis (n = 1376,104)	56.0%	53.1%	20.6%
Psoriasis (n = 1304,624)	47.0%	51.2%	19.3%
Psoriatic Arthritis (n = 373,681)	51.0%	52.9%	20.9%
Systemic Lupus Erythematosus (n = 369,014)	50.6%	40.1%	15.5%
Sjögren’s Syndrome (n = 259,128)	48.2%	49.0%	14.1%
Sarcoidosis (n = 257,563)	57.4%	53.2%	25.8%
Polymyalgia Rheumatica (n = 249,671)	70.1%	70.6%	24.5%
Ankylosing Spondylitis (n = 160,997)	46.1%	47.2%	17.1%
Antiphospholipid Syndrome (n = 137,279)	51.0%	46.8%	18.2%
Scleroderma (n = 94,536)	48.5%	45.9%	12.7%
Giant Cell Arteritis (n = 93,117)	69.1%	66.7%	29.0%
Total (All Diagnoses) (n = 4066,153)	52.1%	51.4%	19.6%

Across all rheumatologic conditions, of patients with diabetes, the mean hemoglobin A1c was 7.1%. The mean LDL-C level was 98 mg/dL, similar across all rheumatologic diagnoses. Among patients with a diagnosis of dyslipidemia, 22.7% were not prescribed any lipid-lowering therapy during the study period.

**Fig. 1:**
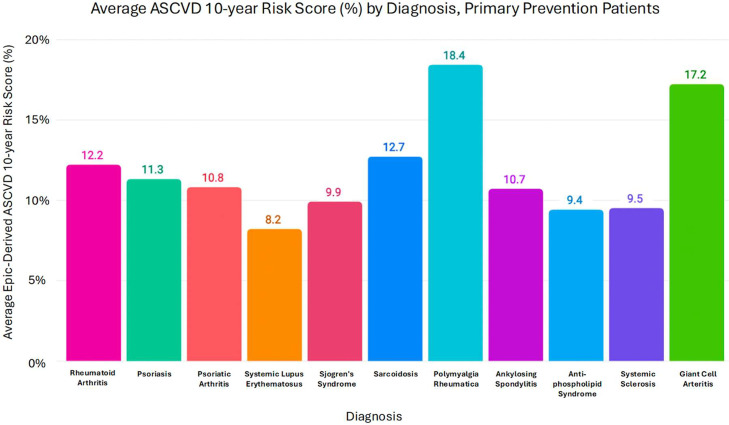
Average 10-year ASCVD Risk score, stratified by diagnosis. Major adverse cardiac events (MACE) and Age <40 or >75 excluded. MACE was defined as myocardial infarction or cerebrovascular disease (TIA/stroke).

For ASCVD risk analysis, 1.4 million patients were excluded given age <40 or >75 years or prior MACE. Of the remaining 2.7 million patients across all diagnoses, 43.0% of patients had a 10-year ASCVD risk <7.5%. 47.5% of patients met criteria for elevated ASCVD risk (≥7.5%). Average ASCVD 10-year risk scores varied across rheumatologic diagnoses, ranging from 8.2% (SLE) to 18.4% (PMR) (Fig. 1).

**Fig. 2:**
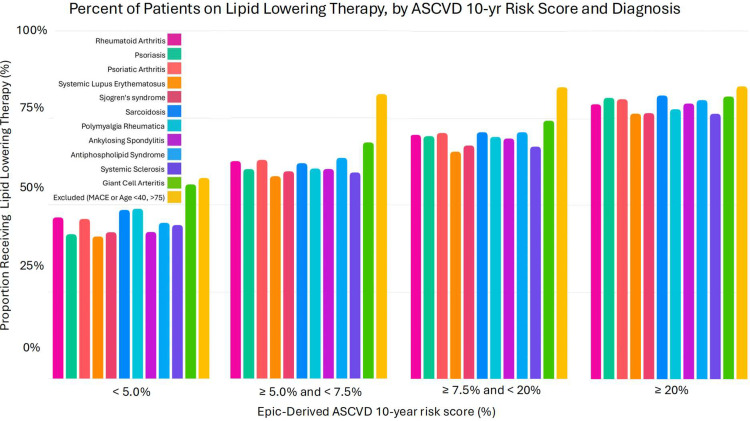
Lipid-lowering prescription patterns in primary prevention patients, stratified by ASCVD 10-year risk score and rheumatologic diagnosis.

Lipid-lowering prescription rates improved with higher ASCVD risk strata yet remained suboptimal. 22.7% of patients with dyslipidemia were not on lipid-lowering therapy. Among those with ASCVD risk 7.5–20%, 28.5% were not on lipid-lowering therapy. For ASCVD risk >20%, 19.4% were not on lipid-lowering therapy. Lipid lowering prescription patterns are outlined (Fig. 2).

**Fig. 3:**
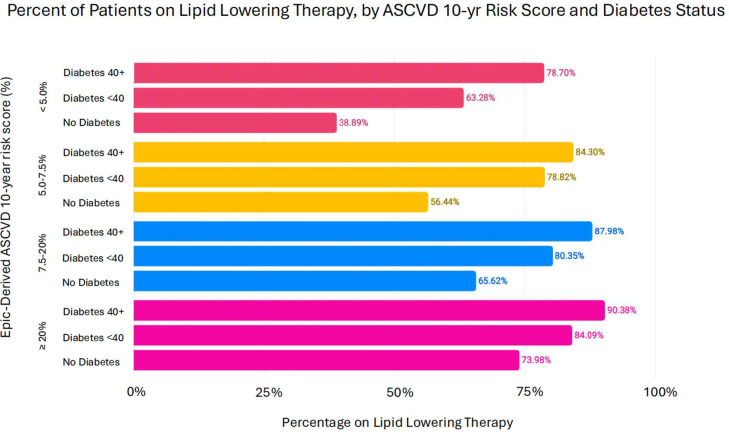
Lipid-lowering prescription patterns stratified by diabetes status and ASCVD 10-year risk score. All ages and MACE status included.

**Fig. 4:**
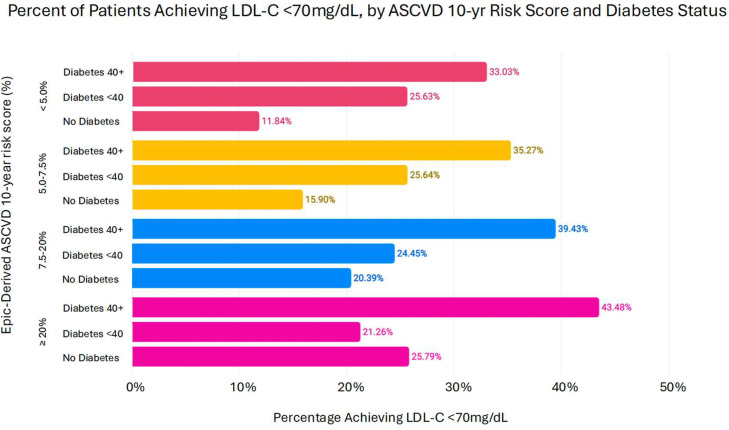
Percentage of patients achieving optimal serum LDL-C levels, by ASCVD 10-year risk score and diabetes status. All ages and MACE status included.

**Fig. 5:**
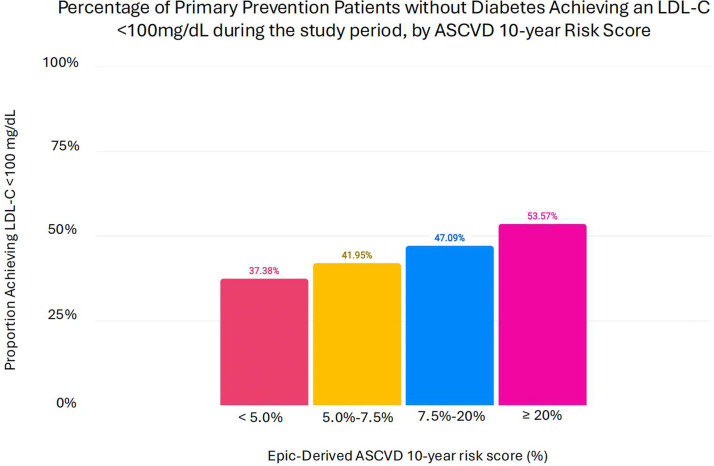
Percentage of primary prevention patients without diabetes achieving LDL-C levels <100mg/dL at any time during the study period, by ASCVD 10-year risk score. Major adverse cardiac events (MACE) and Age <40 or >75 excluded. MACE was defined as myocardial infarction or cerebrovascular disease.

**Fig. 6:**
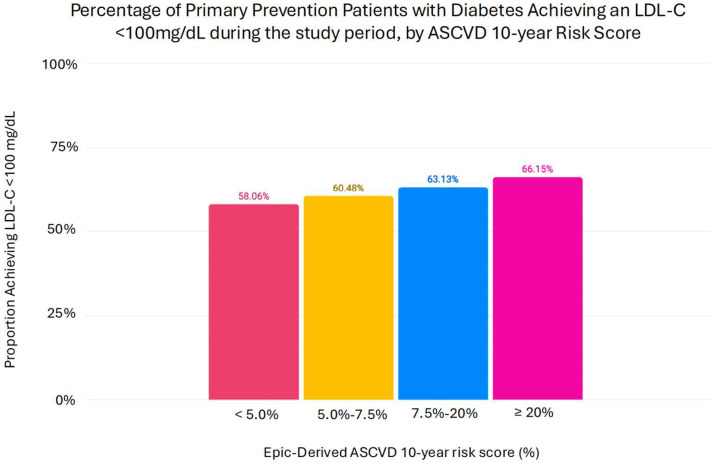
Percentage of primary prevention patients with diabetes achieving LDL-C levels <100mg/dL at any time during the study period, by ASCVD 10-year risk score. Major adverse cardiac events (MACE) and Age <40 or >75 excluded. MACE was defined as myocardial infarction or cerebrovascular disease.

When stratified by diabetes status, lower prescription rates of lipid-lowering therapy were noted in patients with and without diabetes under the age of 40 (Fig. 3). Further, the percentage of patients with a documented LDL-C < 70 mg/dL in the group with ASCVD 10-year risk >20% was only 43.5% of patients with diabetes over age 40 (Fig. 4). When considering a more lenient LDL-C goal of <100mg/dL, only 47.1% of patients without diabetes and ASCVD 10-year risk 7.5–20% achieved this at any time during the study period (Fig. 5 and Fig. 6).

**Fig. 7:**
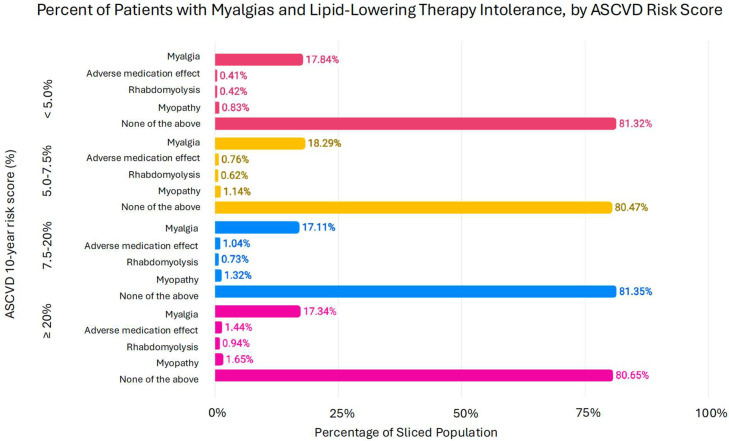
Prevalence of myalgias and intolerance to lipid-lowering therapy, stratified by ASCVD 10-year risk score and musculoskeletal diagnosis. Lipid-lowering intolerance included rhabdomyolysis, adverse effects of or poisoning related to lipid-lowering medication, and myopathy.

**Fig. 8:**
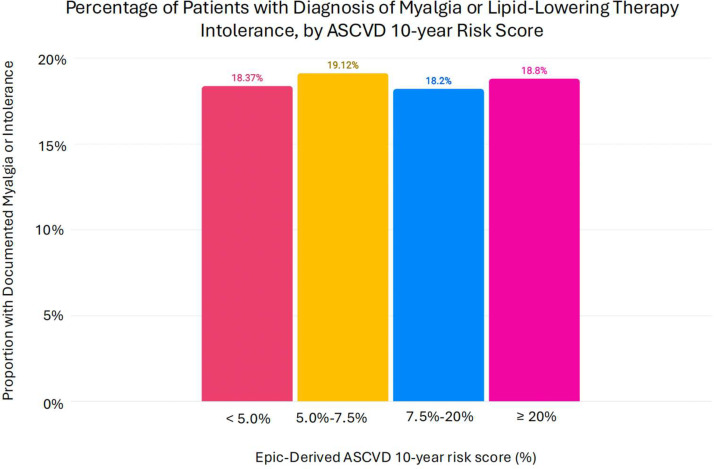
Prevalence of myalgias and intolerance to lipid-lowering therapy, stratified by ASCVD 10-year risk score. Lipid-lowering intolerance included rhabdomyolysis, adverse effects of or poisoning related to lipid-lowering medication, and myopathy.

Myalgias were commonly documented across all ASCVD risk strata and rheumatologic diagnoses (Figs. 7& 8). However, severe muscle-related events were rare (incidence of rhabdomyolysis or a specifically coded adverse reaction to lipid-lowering agent was low across all risk categories, with highest prevalence observed in the ASCVD risk >20% group [1.44%]). Although the dataset did not permit differentiation between inflammatory disease–related musculoskeletal symptoms and statin-associated myalgias, >80% within each ASCVD risk stratum had no documented myalgias, myopathy, rhabdomyolysis, or adverse lipid-lowering reaction during the study period.

**Fig. 9:**
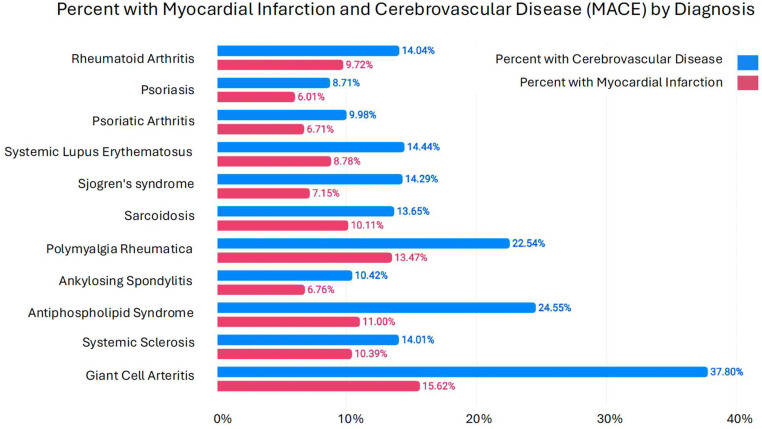
Percent of Cardio-Rheumatologic Cohort with MACE (major adverse cardiac events) defined as myocardial infarction or cerebrovascular disease.

Cardiovascular events were prevalent in the study population, with 8.4% of patients having a diagnosis of myocardial infarction and 13.0% of patients carrying a diagnosis of cerebrovascular disease (Fig. 9).

## 4 Discussion

In this analysis of > 4 million individuals with rheumatologic disease from the EPIC COSMOS database, we report frequently coprevalent cardiometabolic risk factors juxtaposed with low rates of guideline-directed CVD primary prevention goal attainment. Nearly half of eligible patients met criteria for elevated 10-year ASCVD risk (≥7.5%), yet lipid-lowering therapy use and LDL-C goal attainment remained suboptimal. Despite the well-established association between rheumatologic conditions and elevated CVD risk, our findings highlight significant gaps between recommended prevention strategies and prescription thereof.

Consistent with prior literature demonstrating a 1.5–2-fold excess CV risk in conditions such as RA and SLE, our findings confirm the high burden of coprevalent hypertension, dyslipidemia, and diabetes in patients with rheumatologic conditions[1,2]. Although lipid-lowering prescription rates increased with higher estimated risk, suggesting that clinicians are responsive to risk stratification, nearly 20% of patients with ASCVD risk >20% were not prescribed lipid-lowering therapy at any time during the analytic period.

While longitudinal goal attainment could not be ascertained, we queried data to determine rate of LDL-C < 70 mg/dL and <100 mg/dL achievement at any point during the study period. The 2026 lipid guidelines give a 2a recommendation for LDL goal <100 mg/dL in intermediate and high-risk patients, including considering the risk enhancing factors of systemic inflammatory conditions [5]. However, in our cohort, only about 1 in 2 intermediate to high risk primary prevention patients without diabetes reached an LDL-C nadir of <100 mg/dL.

These findings underscore that lipid-lowering therapy remains under-prescribed, particularly among patients with moderate ASCVD risk. Paradoxically we also note suboptimal prescription rates of lipid-lowering therapy among nondiabetic individuals, even at comparable ASCVD risk levels. Given that approximately 80% of the cohort did not carry a diagnosis of diabetes, this group of patients with a rheumatologic condition, but without diabetes, represents the largest absolute number of untreated high-risk patients. This pattern may reflect a cognitive bias in risk assessment, whereby diabetes serves as a stronger trigger for statin initiation than calculated ASCVD risk, despite systemic inflammatory disease being considered risk-enhancers.

One potential barrier to intensification is medication intolerance. Patients with systemic inflammatory diseases more frequently report baseline musculoskeletal symptoms, perhaps contributing to clinician reluctance in initiating/escalating lipid-lowering therapy. However, our findings suggest that documented severe statin-associated toxicity was rare. (>80% of patients across all ASCVD risk categories had no recorded muscle-related adverse events). In this context, perceived intolerance may contribute to therapeutic inertia, underscoring the need for statin rechallenge protocols, alternative dosing strategies, and incorporation of non-statin agents in patients with true intolerance.

Prior studies have reported an increased risk of CVD in patients with rheumatologic conditions, yet treatment gaps persist [6,7]. Our findings support engagement in multidisciplinary care pathways, standardized risk assessment protocols embedded within rheumatology clinics, and automated electronic health record prompts to improve implementation of preventive therapies.

Finally, the high prevalence of myocardial infarction and cerebrovascular disease (>750,000 patients) further underscores the sequelae of cumulative inflammatory and metabolic risk. These findings support that cardiovascular prevention should be integrated into routine management of all systemic inflammatory diseases. Given the scale of this cohort, even modest improvements in lipid optimization could translate to meaningful reductions in myocardial infarction and stroke risk.

## 5 Limitations

This study should be interpreted in the context of several limitations inherent to retrospective analyses using administrative EMR data. The data represents a cross-sectional snapshot of the patient cohort rather than a longitudinal assessment of risk factor management over time. While the study provides insight into the persistent gap between guideline-recommended and real-world practice, it does not account for drivers of provider decision-making, patient preferences, or contraindications that may affect treatment choices. Medication prescriptions do not confirm adherence, and LDL-C measurements reflect recorded values rather than standardized follow-up intervals. The variability in healthcare settings may introduce inconsistencies in the observed rates of CVD prevention goal attainment. Additionally, the definition of lipid-lowering intolerance was based on ICD coding.

## 6 Conclusions

In the largest U.S. cohort of patients with rheumatologic disease reported to date, cardiovascular risk factor burden is high and guideline-directed lipid management remains suboptimal. Although lipid-lowering prescriptions improve as ASCVD risk rises, substantial gaps persist, even among the highest-risk individuals. These findings highlight the need for multidisciplinary models and implementation strategies aimed at translating established prevention guidelines into routine care.

Future studies should explore interventions that enhance provider awareness of CVD risk in autoimmune diseases, promote interdisciplinary collaboration between rheumatologists and cardiologists, and develop targeted prevention strategies tailored to the unique inflammatory burden in this population.

**Figure:**
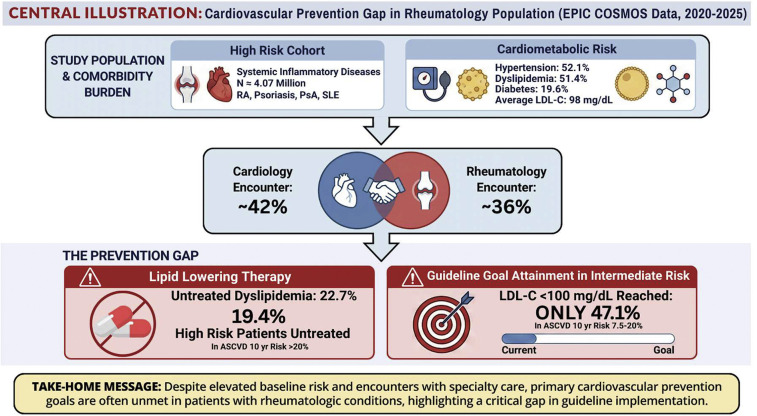
**Central Illustration**.

## Author agreement

Cardiovascular risk goal attainment in the United States Rheumatologic Population

Drs. Granstaff, Grodzinsky and medical students Jiang and Nallagatla certify that all authors have seen and approved the final version of the manuscript being submitted. We warrant that the article is the authors' original work, and that it hasn't received prior publication and isn't under consideration for publication elsewhere.

## CRediT authorship contribution statement

**Kaitlyn Granstaff:** Writing – review & editing, Writing – original draft, Project administration, Methodology, Investigation, Formal analysis, Data curation, Conceptualization. **Sarah Jiang:** Writing – review & editing, Visualization, Resources, Project administration, Methodology, Investigation, Data curation. **Sanjana Nallagatla:** Writing – review & editing, Writing – original draft, Visualization, Project administration, Methodology, Investigation, Data curation. **Anna Grodzinsky:** Writing – review & editing, Writing – original draft, Validation, Supervision, Project administration, Methodology, Investigation, Funding acquisition, Formal analysis, Data curation, Conceptualization.

## Declaration of competing interest

The authors declare that they have no known competing financial interests or personal relationships that could have appeared to influence the work reported in this paper.
